# Single-cell RNA sequencing reveals hub genes of myocardial infarction-associated endothelial cells

**DOI:** 10.1186/s12872-024-03727-z

**Published:** 2024-01-24

**Authors:** Hao Wang, Liping Dou

**Affiliations:** 1 Department of Cardiovascular Medicine, Zhejiang Greentown Cardiovascular Hospital, No.409 Gudun Road, Hangzhou, 310000 Zhejiang China; 2 Department of Geriatrics, The Second Affiliated Hospital of Zhejiang Chinese Medical University, No. 318 Chaowang Road, Hangzhou, 310005 Zhejiang China

**Keywords:** Myocardial infarction, Endothelial cells, scRNA-seq, Hub genes

## Abstract

**Background:**

Myocardial infarction (MI) is a cardiovascular disease that seriously threatens human health. Dysangiogenesis of endothelial cells (ECs) primarily inhibits recovery from MI, but the specific mechanism remains to be further elucidated.

**Methods:**

In this study, the single-cell RNA-sequencing data from both MI and Sham mice were analyzed by the Seurat Package (3.2.2). The number of ECs in MI and Sham groups were compared by PCA and tSNE algorithm. FindMarkers function of Seurat was used to analyze the DEGs between the MI and Sham groups. Then, the ECs was further clustered into 8 sub-clusters for trajectory analysis. The BEAM was used to analyze the branch point 3 and cluster the results. In addition, the DEGs in the microarray data set of MI and Sham mice were cross-linked, and the cross-linked genes were used to construct PPI networks. The key genes with the highest degree were identified and analyzed for functional enrichment. Finally, this study cultured human umbilical vein endothelial cells (HUVECs), established hypoxia models, and interfered with hub gene expression in cells. The impact of hub genes on the migration and tube formation of hypoxic-induced HUVECs were verified by Wound healing assays and tubule formation experiments.

**Results:**

The number and proportion of ECs in the MI group were significantly lower than those in the Sham group. Meantime, 225 DEGs were found in ECs between the MI and Sham groups. Through trajectory analysis, EC4 was found to play an important role in MI. Then, by using BEAM to analyze the branch point 3, and clustering the results, a total of 495 genes were found to be highly expressed in cell Fate2 (mainly EC4). In addition, a total of 194 DEGs were identified in Micro array dataset containing both MI and Sham mice. The hub genes (Timp1 and Fn1) with the highest degree were identified. Inhibiting Timp1 and Fn1 expression promoted the migration and tube formation of HUVECs.

**Conclusions:**

Our data highlighted the non-linear dynamics of ECs in MI, and provided a foothold for analyzing cardiac homeostasis and pro-angiogenesis in MI.

**Supplementary Information:**

The online version contains supplementary material available at 10.1186/s12872-024-03727-z.

## Introduction

 Cardiovascular disease is the most common cause of death in the world [1]. Myocardial infarction (MI) is a major cardiac syndrome and a main inducing factor of cardiovascular disease-related deaths [2]. During MI, impaired myocardial function leads to reduced cardiac contractility, ultimately resulting in heart failure [3]. The occurrence of MI is primarily attributed to myocardial ischemia and hypoxia after coronary artery occlusion. Previous studies have shown that upregulation of angiogenesis attenuates ventricular remodeling and improves cardiac function after MI [4, 5]. Therefore, therapeutic angiogenesis is a promising approach in ischemic myocardium.

Endothelial cells (ECs) is the most abundant cell type in the adult heart and vasculature [9]. ECs line the inner lining of blood vessels and are essential for blood supply homeostasis and cardiac protection. After MI, ECs further inhibit the recovery of cardiac function through several cell death processes that affect coronary artery vessel density in ischemic areas [10]. On the other hand, ECs have been found to play important roles in maintaining homeostasis by promoting blood vessel formation and building blood vessel barriers [11]. Neovascularization is a key process in cardiac regeneration [12]. According to previous studies on the angiogenesis of ECs, E2F1 inhibits VEGF and PlGF upregulation through p53 dependent and independent mechanisms, respectively, thereby limiting apoptosis and neovascularization of cardiac ECs after MIn [13]. VASH1 overexpression can eliminate the invasion and tube formation of ECs induced by hypoxia or ZNF667 overexpression [14]. Lack of SGK1 leads to impaired EC migration and angiogenesis, increased scarring and decreased angiogenesis in vivo after MI [15]. All these findings imply that ECs and ECs-associated genes positively affects the progression of MI.

Recently, with the advent of single-cell RNA-sequencing (scRNA-seq), it is possible to perform transcriptome analysis of large numbers of cells with single-cell resolution, which not only provides specificity analysis of transcriptional signature in individual cells [18], but also offers powerful tools in studying cell heterogeneity [19]. For instance, with the aid of scRNA-seq, the heterogeneity and dynamics of cardiac fibroblasts (CFs) was reported during MI [20]. Additionally, scRNA-seq was also used to identify and characterize the population of Tregs in heart, which provides a basis that Tregs play a protective role in heart diseases including MI [21]. By performing scRNA-seq, Ziwen Li et al [22] demonstrated that the structural integrity of adult heart endothelium after MI was maintained by clonal proliferation of resident ECs in the infarct boundary region. Ten heterogeneous EC states with discrete transcription were also defined, as well as pathways by which each endothelial state might enhance neovascularization and tissue regeneration after ischemic injury. In addition, Monocle 2 is a recently developed algorithm using the reversed graph embedding strategy, which can accurately reconstruct the trajectory of a single cell based on cell differentiation [23]. Thus, sub-clusters of cells in differentiation can be classified by combinatorial single-cell genomics and locus analysis [24].

In this study, scRNA-seq was used to analyze the cell heterogeneity of samples from MI mice. Genes related to the differentiation of ECs were identified. Combined with the differential expression analysis between the MI and the control group, hub genes were obtained. In addition, the effects of hub genes on EC migration and tube formation under hypoxia were studied. Our study suggested the promising therapeutic targets for MI.

## Materials and methods

### Data collection

scRNA-seq and microarray data of MI mice were from the Gene Expression Omnibus (GEO) database (https://www.ncbi.nlm.nih.gov/geo/). In dataset GSE136088 (https://www.ncbi.nlm.nih.gov/geo/query/acc.cgi?acc=GSE136088), the scRNA-seq data of 1 MI sample and 1 Sham sample from the heart cell populations in MI mice and Sham mice were analyzed. In dataset GSE23294 (https://www.ncbi.nlm.nih.gov/geo/query/acc.cgi), the microarray data of 10 MI samples and 10 Sham samples from the left ventricular of mice were analyzed.

### scRNA-seq data processing

Seurat package (3.2.2) was applied for performing scRNA-seq data analysis. After screening (genes detected in < 5 cells, cells with < 300 total detected genes, and low-quality cells with mitochondria-expressed genes ≥ 20% were excluded), the gene expression of the remaining cells was normalized through a linear regression model. The MI and Sham samples were integrated using IntegrateData of Seurat package to eliminate batch effects. Available dimensions with (*P* < 0.05) was determined and dimensionality reduced by principal component analysis (PCA) and the t-distributed stochastic neighbor embedding (tSNE) algorithm. Then, cluster classification analysis was performed. The cell types of cell clusters were annotated in line with cell marker genes obtained from literature review and the CellMarker Database (http://xteam.xbio.top/CellMarker/).

### Differential analysis

For ECs, FindMarkers function of Seurat was used to analyze the differentially expressed genes (DEGs) between the MI and Sham samples (log_2_FC > 0.25, *P* < 0.05, test.use = “Wilcox”). For the GSE23294 dataset, limma was used to analyze the DEGs between the MI and Sham samples.

### Gene set enrichment analysis (GSEA)

GSEA is an effective analytical approach to interpreting the expression profiles of whole genome [25]. In this study, ClusterProfiler was used to conduct GSEA on the DEGs in ECs based on KEGG pathway enrichment.

### Sub-cluster analysis of ECs

ECs was divided into sub-clusters, and the differences between MI and Sham in the distribution of each sub-cluster were analyzed. FindAllMarkers was used to analyze the marker genes in each sub-cluster.

### Trajectory analysis of ECs

Monocle 2 algorithm was used to construct the single-cell pseudotime trajectories of the ECs. Cells in the same differentiation state were projected onto the same branches. Conversely, cells of different differentiation states were located in different branches. Next, genes with differential expression levels were considered as branch-dependent/state-specific genes or marker genes through differential expression analysis.

### Function analysis of the sub-clusters of ECs

Shown by the trajectory analysis, EC4 was quite different from other sub-clusters. As a result, GSEA was performed on EC4 and other sub-clusters, respectively.

### Identification of hub genes

DEGs in ECs, EC4-related genes identified via trajectory analysis, and DEGs of GSE23294 was interested. The clusterProfiler package and the org.Hs.eg.db package were used for performing GO and KEGG enrichment analyses. Additionally, PPI analysis was conducted using the STRING website (Medium confidence = 0.4, https://cn.string-db.org/).

### Cell culture and treatment

Human umbilical vein endothelial cells (HUVECs, American Type Culture Collection) were cultured in DMEM (Gibco-BRL) containing 10% new-born calf serum (Invitrogen Life Technologies) and 1% penicillin-streptomycin (Invitrogen; Thermo Fisher Scientific) at 37 °C with 5% CO_2_ and 21% O_2_ (371; Thermo Fisher Scientific). In order to induce hypoxia, HUVECs were cultured with 1% O_2_, 5% CO_2_ and 94% N_2_ (3131; Thermo Fisher Scientific) for 48 h.

### Cell transfection

In line with the manufacturer’s instructions, tissue inhibitor of matrix metalloproteinase 1 (Timp1) siRNA or control siRNA (JTS scientific company, Wuhan, China), and Fibronectin 1 (Fn1) siRNA or control siRNA (Santa Cruz Biotechnology, Dallas, TX, USA, cat#: sc-29,315) were transfected into cells using lipofectamine 3000 (Invitrogen, MD, USA).

### Western blot

The treated cells were lysed. After determination of the protein concentration, a total of 25 µg protein was separated and transferred to PVDF membranes (Millipore, Bedford, USA). After blocking, the membrane was incubated overnight with the primary antibodies at 4 °C. The proteins were detected using an enhanced chemiluminescence (ECL) system. The antibodies used are as follows: Rabbit antibodies against TIMP1, Fn1, and GAPDH (ab109125, ab2413, and ab181602, 1:2000, Abcam, Cambridge, MA, USA).

### Wound healing assay

After the cells in 35-mm dishes formed a 100% confluent monolayer, a 100-µL tip was used to stab the cells. The results of wound healing were observed via a microscope at 0 and 24 h.

### Tube formation assay

The treated HUVECs (500 µL/well) were added into a 24-well plate coated with Matrigel matrix (BD Biosciences). After 4 h, calcein solution (Solarbio, Beijing, China) was added at 37℃ for 15–30 min. The cells were observed under a fluorescence microscope (Nikon TiS, Tokyo, Japan) with an excitation wavelength of 490 nm and an emission wavelength of 515 nm. The tube formation results were observed under an inverted microscope and analyzed using the Image J software.

### Statistical analysis

GraphPad Prism 8.0 (GraphPad Software, USA) was used for performing statistical analysis, and the results were expressed as mean ($\stackrel{-}{\text{x}}$) ± standard deviation (SD). The two-tailed unpaired Student’s *t*-test or one-way analysis of variance were used for making group comparisons. A *P* < 0.05 was considered significant.

## Results

### High cell heterogeneity revealed by identifying 16 cell clusters in MI using scRNA-seq data

**Fig. 1:**
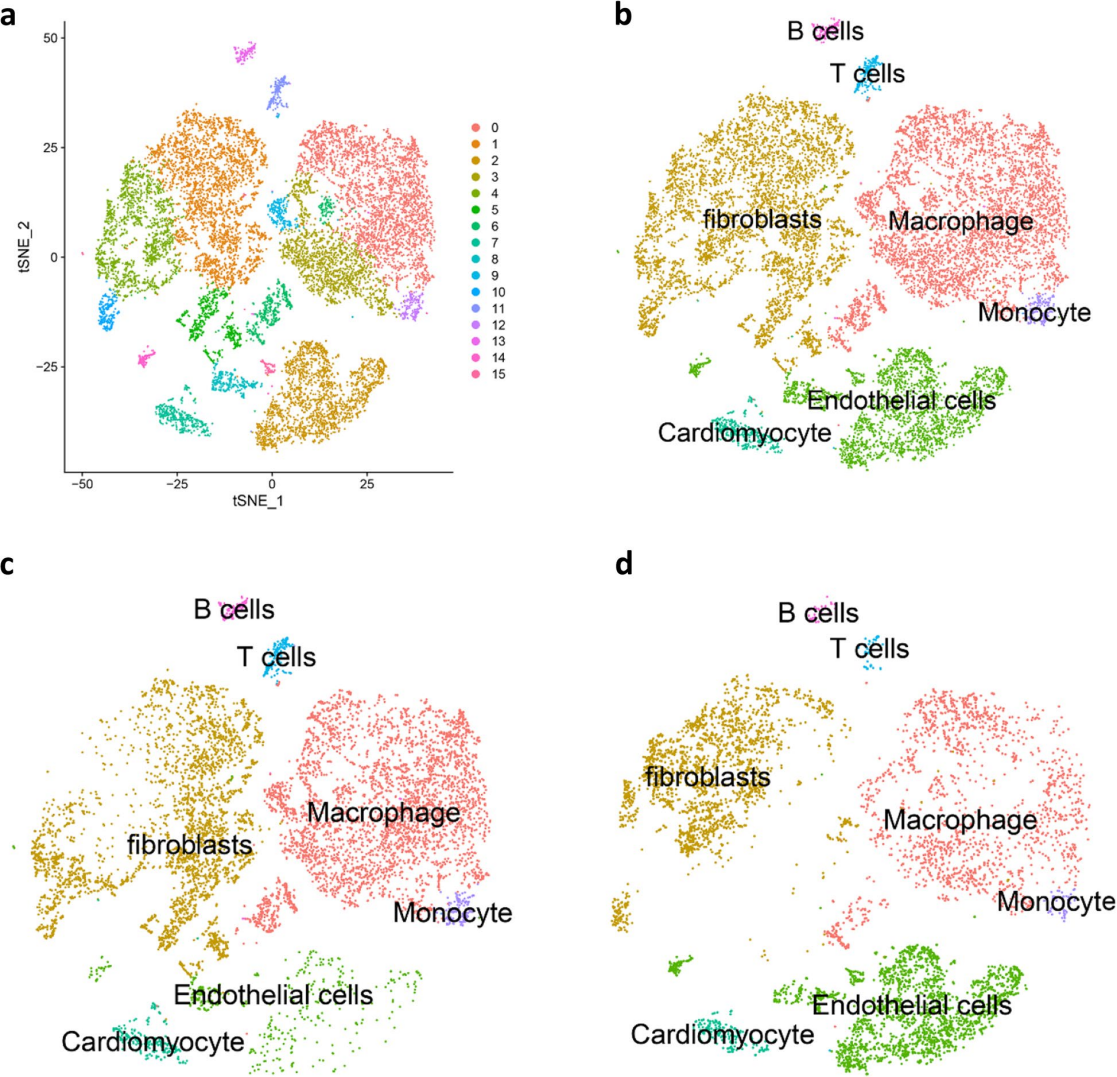
High cell heterogeneity revealed by identifying 16 cell clusters in MI using scRNA-seq data. **a** The tSNE algorithm was applied for dimensionality reduction with the 20 PCs, and 16 cell clusters were successfully classified. **b** 16 cell clusters were annotated by CellMarker according to the composition of the marker genes. Cell clusters in the MI group (**c**) and the Sham group (**d**) were annotated by CellMarker according to the composition of the marker genes

After initial quality control and batch effect correction, single-cell transcriptomes in 8494 cells (MI samples) and 6257 cells (Sham samples) were obtained. Then, PCA was conducted for dimensionality reduction and investigation of cellular composition. Afterwards, cells were classified into 16 separate clusters using the tSNE algorithm (Fig. 1a). Based on the expression patterns of these marker genes, these clusters were annotated by CellMarker (Fig. 1b-d) as macrophage, fibroblasts, endothelial cells, cardiomyocyte, monocytes, T and B cells. The number and proportion of ECs in the MI group (446 ECs, 5.25%) were significantly lower than those in the Sham group (2189 ECs, 34.98%).

### Identification of DEGs and sub-clusters of ECs

**Fig. 2:**
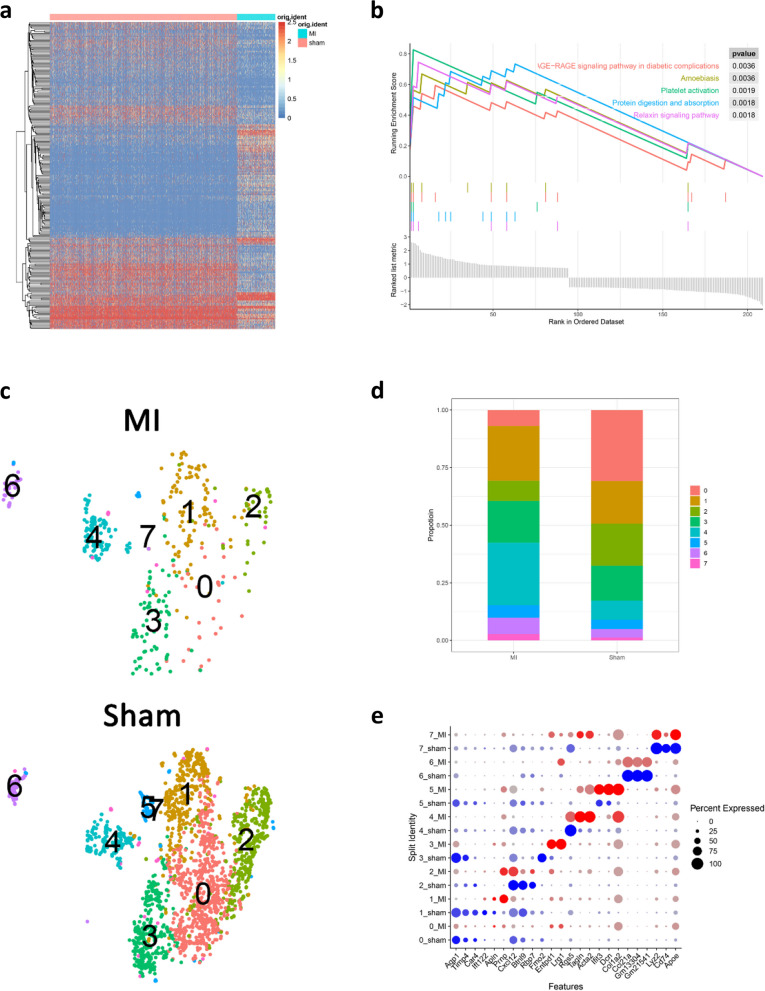
Identification of DEGs and sub-clusters of ECs. **a** Heatmap of DEGs in ECs. **b** GSEA enrichment analysis of DEGs. **c** Sub-clusters of ECs in the MI and Sham groups. **d** Proportion of EC sub-clusters in the MI and Sham groups. **e** Top 3 marker genes of EC sub-clusters

Next, we analyzed the DEGs in ECs between the MI and Sham groups using FindMarkers, and identified a total of 225 DEGs (96 upregulated DEGs and 129 downregulated DEGs) (Fig. 2a). These DEGs were found to be enriched in the AGE − RAGE signaling pathway in diabetic complications, protein digestion and absorption, and platelet activation (Fig. 2b). ECs were further clustered into 8 sub-clusters (EC0-EC7, Fig. 2c-d). Moreover, the proportion of the sub-clusters of ECs in the MI and Sham groups was significantly different, and the proportion of EC0 in the MI group (6.95%) was significantly lower than that in the Sham group (30.79%), while the proportion of EC4 in the MI group (27.13%) was higher than that in the Sham group (8.36%). In addition, the marker genes of each sub-clusters were analyzed by FindAllMarkers function, and the top 3 marker genes were displayed by dotplot (Fig. 2e).

### Trajectory analysis of ECs

**Fig. 3:**
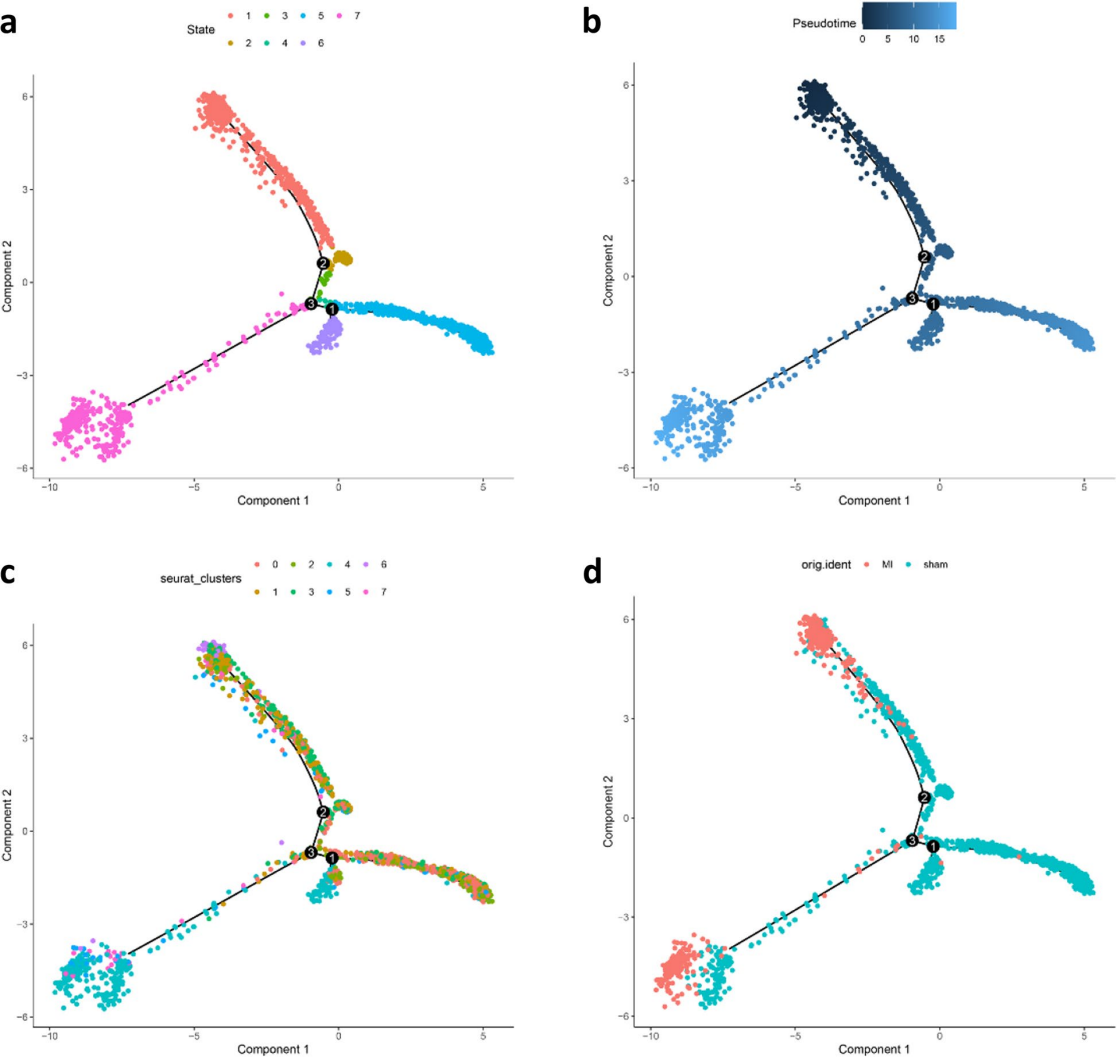
Trajectory analysis of EC sub-clusters according to state (**a**), pesudotime (**b**), sub-clusters (**c**), and condition (**d**)

**Fig. 4:**
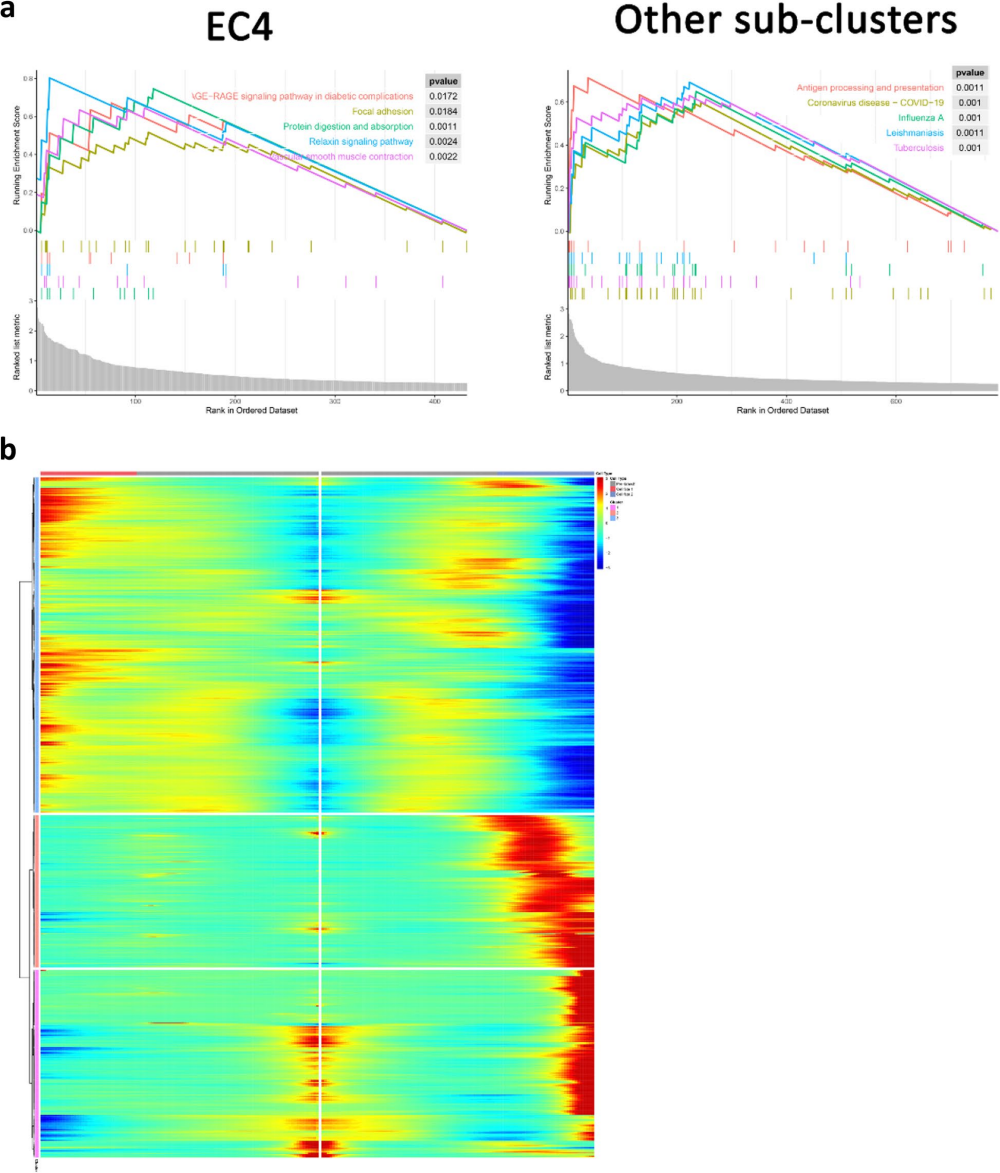
GSEA and BEAM analysis of ECs. **a** GSEA of EC4 and other sub-clusters. **b** Branch-dependent genes in BEAM analysis, with pre-branch representing cells in state 1, 2 and 3, cell fate 1 representing cells in state 3 and 4, and cell fate 2 representing cells in state 7 (EC4)

To reveal the changes of different EC sub-clusters during MI, we performed trajectory analysis of ECs by state, pesudotime, sub-cluster, and condition (Fig. 3a-d). The results showed that ECs could be divided into State1-7. State7 was mainly EC4 sub-cluster, and EC4 was primarily distributed in State1 and State7 in MI. Therefore, we speculated that EC4 plays an important role in MI. Then, GSEA enrichment analysis was performed on EC4 and other sub-clusters (Fig. 4a). EC4 was mainly enriched in the AGE-RAGE signaling pathway in diabetic complications, relaxin signaling pathway, vascular smooth muscle contraction, and focal adhesion. Other sub-clusters were enriched in antigen processing and presentation, coronavirus disease-COVID-19, influenza A and other pathways. Then, branch-dependent genes of Branch point 3 were analyzed by BEAM and clustered. The results showed that these genes could be clustered into 3 clusters. Cluster 1 and cluster 2 contained 495 genes, which were highly expressed in cell fate 2 (State 7) (Figs. 3a and 4b).

### Identification of DEGs in GSE23294

**Fig. 5:**
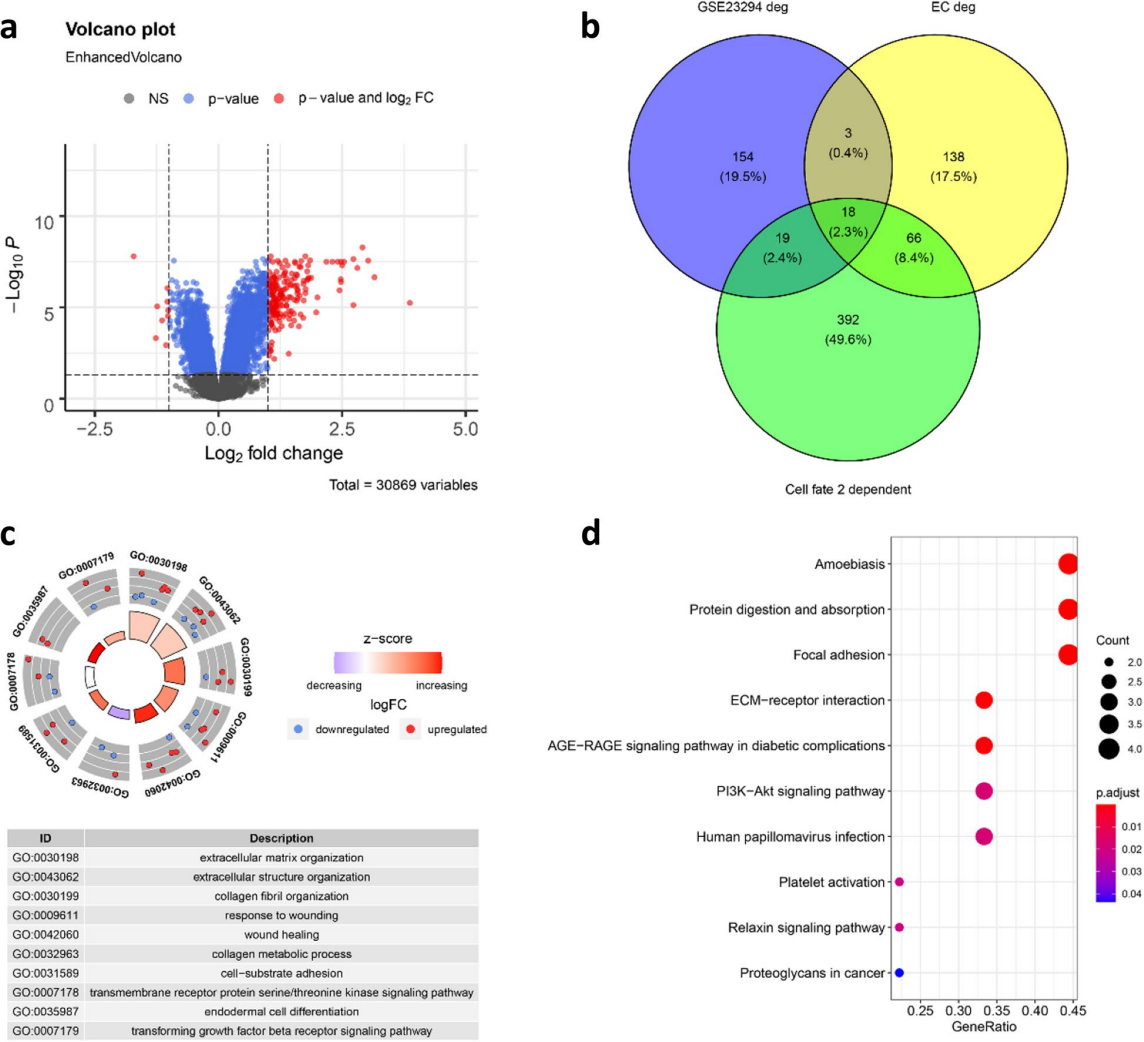
Identification of DEGs in GSE23294. **a** The volcano plot of DEGs in GSE23294. **b** Venn diagram of the intersected genes. The GO (**c**) and KEGG (**d**) enrichment analysis of the 18 intersected genes

Meantime, the DEGs between MI and Sham groups in GSE23294 were analyzed by Limma, after which 194 DEGs (185 upregulated and 9 downregulated) were identified (Fig. 5a). The cell fate 2 dependent genes in trajectory analysis, DEGs in ECs, and DEGs in GSE23294 were intersected, and a total of 18 genes were identified (Fig. 5b). GO and KEGG analyses were performed on these 18 genes, and these genes were revealed to be related to focal adhesion, protein digestion and absorption as well as other functions and pathways (Fig. 5c-d).

### PPI network construction and identification of MI-related hub genes

**Fig. 6:**
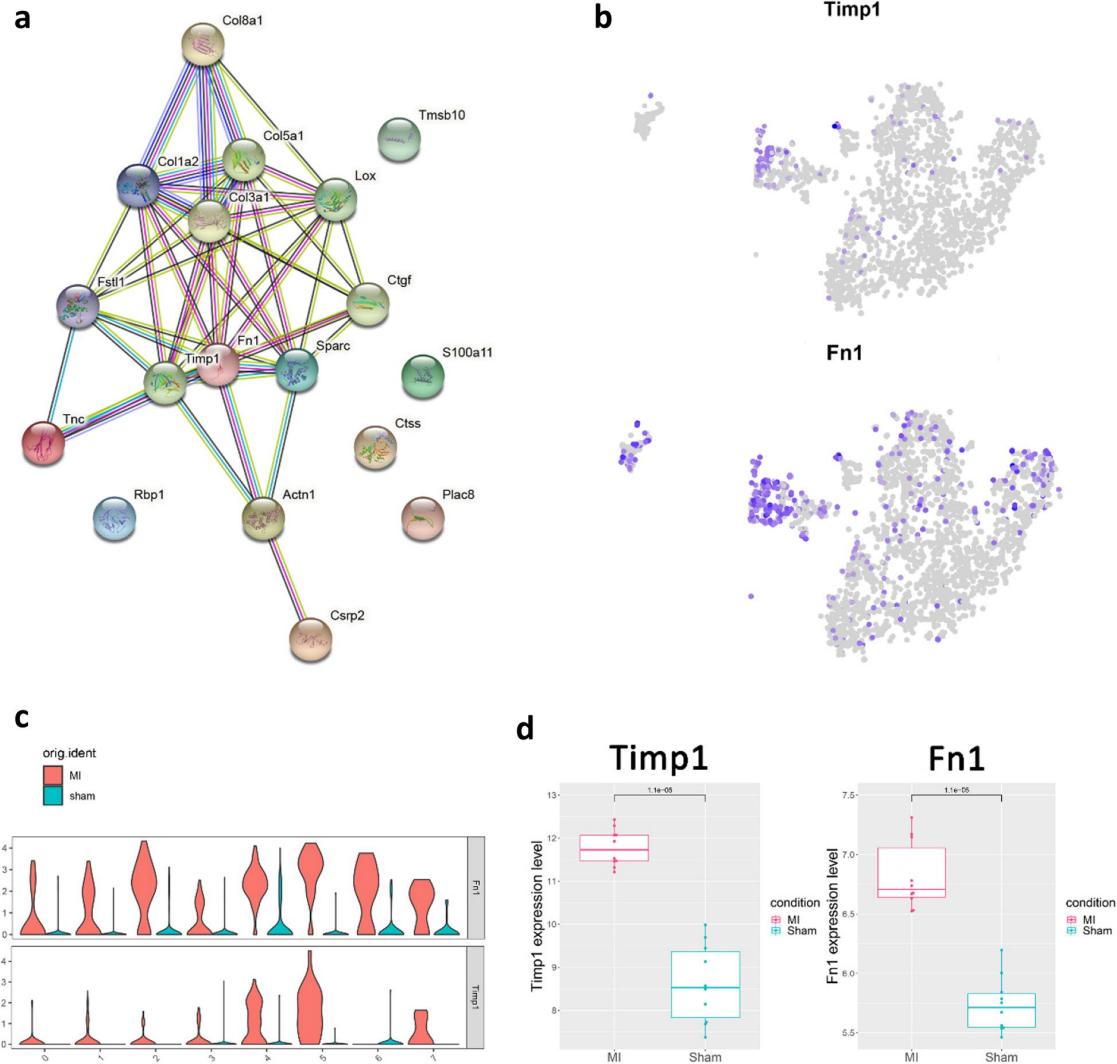
Construction of a PPI network and identification of MI-related hub genes. **a** PPI network of 18 intersected genes using the STRING database. **b** Expression levels of Timp1 and Fn1 in each sub-cluster were plotted onto the t-SNE map. **c** Violin plots of Timp1 and Fn1 across the sub-clusters. **d** Boxplot plots of Timp1 and Fn1 in the MI and Sham group in GSE23294

A PPI network was constructed using the STRING database for the 18 identified genes mentioned above (Fig. 6a), with 13 nodes and 46 edges. The degrees of genes in the PPI network were listed in Table 1. We defined the 2 genes, Timp1 and Fn1 with the highest degree as MI-related hub genes. Figure 6b-c showed that they were mainly distributed in EC4 and were significantly highly expressed in EC4. In GSE23294, compared with the Sham group, Timp1 and Fn1 were also significantly highly expressed in the MI group (Fig. 6d).

**Table 1:** Gene degrees in PPI

Symbol	Number Of Edges
Timp1	10
Fn1	10
Sparc	9
Col1a2	9
Col5a1	9
Lox	9
Col3a1	9
Fstl1	8
Ctgf	7
Actn1	4
Col8a1	4
Tnc	3
Csrp2	1

#### Timp1 and Fn1 were upregulated in hypoxia-induecd HUVECs and inhibited cell migration and tube formation

**Fig. 7:**
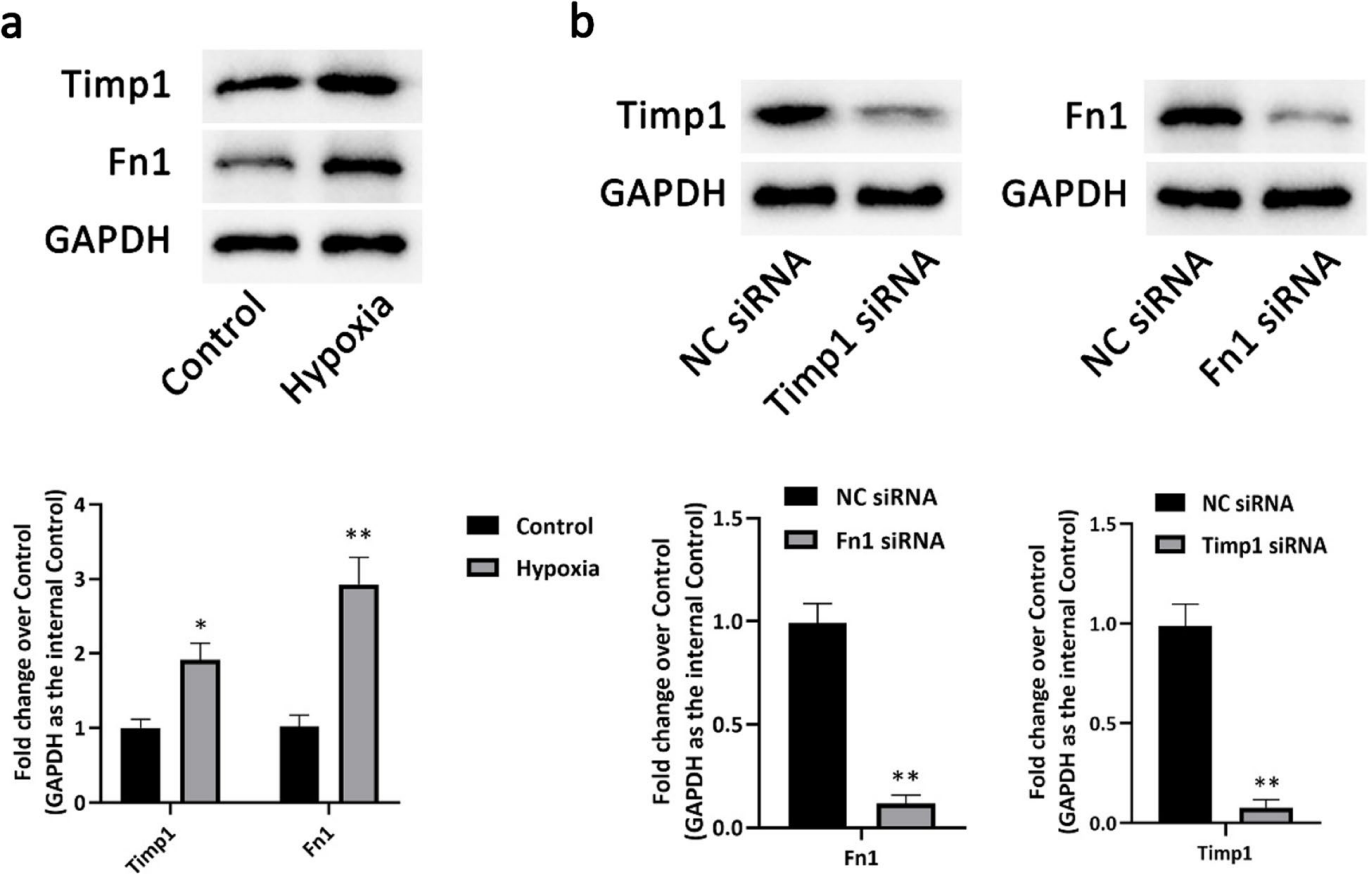
Timp1 and Fn1 were upregulated in hypoxia-induecd HUVECs. **a** The expressions of Timp1 and Fn1 in HUVECs induced by hypoxia detected by Western blot. **b** The expressions of Timp1 or Fn1 in HUVECs transfected with siRNA of Timp1 or Fn1 detected by Western blot. ^*^ *P* < 0.05 compared with the Control group; ^**^ *P* < 0.01 compared with the Control group

**Fig. 8:**
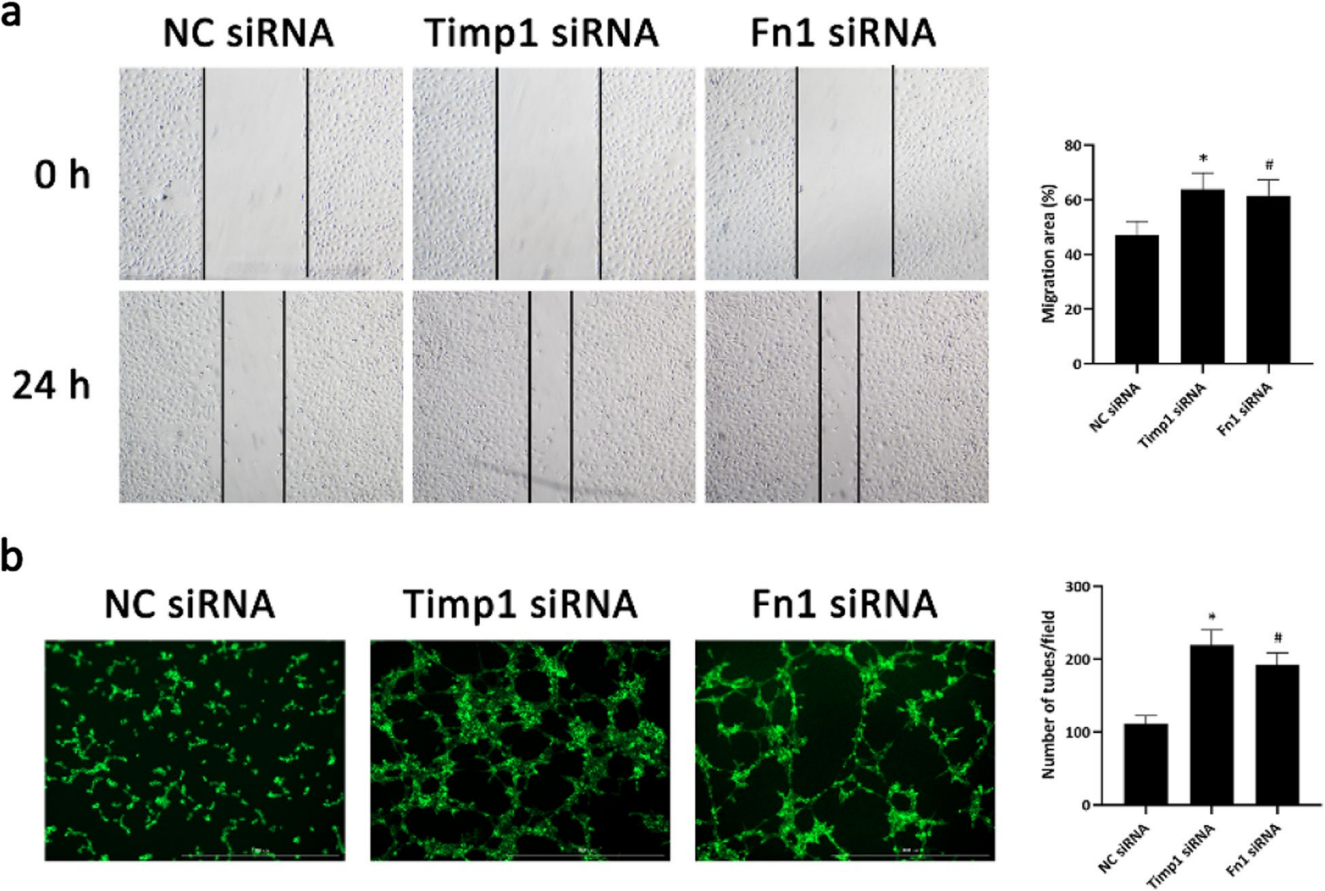
Timp1 and Fn1 inhibited cell migration and tube formation of HUVECs. **a** Wound healing assays were used to examine the migration of HUVECs transfected with siRNA of Timp1 or Fn1. **b** Tube formation of HUVECs after transfection with the siRNA of Timp1 or Fn1. ^*^ *P* < 0.05 Timp1 siRNA compared with the NC siRNA group; ^#^ *P* < 0.05 Fn1 siRNA compared with the Control group

To investigate the roles of Timp1 and Fn1 on ECs, we induced cell injury through hypoxia. Western blot results showed that Timp1 and Fn1 were markedly upregulated after treatment with hypoxia (Fig. 7a). Next, siRNA of Timp1 and Fn1 were transfected before hypoxia (Fig. 7b). Wound-healing experiments illustrated that the migration of HUVECs was markedly increased by Timp1 siRNA and Fn1 siRNA transfection (Fig. 8a). Matrigel-based angiogenesis assay revealed that the tube formation in the hypoxia group was significantly increased after Timp1 siRNA and Fn1 siRNA transfection (Fig. 8b).

## Discussion

This study characterized the heterogeneity of ECs and other cells in the heart tissues of MI and Sham mice through scRNA-seq, and obtained the DEGs of ECs between the MI and Sham groups. Clustering analysis was performed to identify and analyze EC sub-clusters. Then, through trajectory analysis, EC4 was revealed to have close association with MI, and cell fate-dependent genes were obtained by BEAM. At the same time, DEGs in another Micro array dataset containing both MI and Sham mice were analyzed, and interested with the above-mentioned genes. Additionally, hub genes (Timp1 and Fn1) were identified through PPI network construction. They were shown to be enriched in wound healing through enrichment analysis of the functional pathways about the above intersected genes. To study the role of Timp1 and Fn1 in ECs and MI, we treated MI in vitro by inducing hypoxia in ECs, which is the key angiogenic factor. Under hypoxia, Timp1 and Fn1 were significantly upregulated in HUVECs. Silencing of Timp1 and Fn1 increased the migration and angiogenesis of hypoxia-treated HUVECs. Taken together, deficiency of Timp1 and Fn1 may improve MI by promoting angiogenesis.

Timp1 is a member of the tissue inhibitor of matrix metalloproteinase family, and its function is to mediate the turnover of extracellular matrix [26]. Studies have shown that Timp1 is involved in pathological processes [27], and is a key antiangiogenic factor [28]. In addition, recombinant Timp1 was revealed to inhibit the migration and tube formation of HUVECs [29]. The research of Jung-Kyun Choi et al. [30] shows that TIMP1 knockdown in FECS-Ad inhibited angiogenesis and muscle regeneration induced by FECS-Ad transplanted into ischemic mouse tissue. Consistent with the findings of previous literature, Timp1 knockdown was revealed to promote the migration and tube formation of HUVECs, but its specific mechanism is still unclear. Fibronectin 1 (Fn1), as a member of the glycoprotein family [31], is essential for cell growth, differentiation, and plays an important part in wound healing [32]. As reported, VEGF regulated Fn1 to affect the proliferation, migration and angiogenesis of HUVECs [33]. For example, the elimination of Fn1 leads to inhibition of tumor angiogenesis [34]. In addition, Tianyi Chen et al. [35] found that Fn1 is located in human skin ECs and promotes the migration and tube formation of HUVECs. Restricted blood supply can lead to irreversible damage to myocardial cells, eventually leading to ventricular failure [36]. Angiogenesis, the process by which new blood vessels form from the existing vasculature, is often inhibited in MI [37]. Therefore, in recent years, scholars have conducted many studies on promoting angiogenesis to stimulate the recovery of the microvascular system. Rescue of damaged angiogenesis is a prerequisite for developing new treatments. Both Timp1 and Fn1 have demonstrated inhibitory effects on angiogenesis in multiple studies. These studies indirectly prove the important role of inhibitingTimp1 and Fn1 in improving MI.

The limitation of the current study is that only one MI and one Sham sample were analyzed by scRNA-seq. Although the reliability of scRNA-seq results was supported by the expression of hub genes in GEO datasets and HUVECs, however, due to the limitation of sample size in this study, we expect to obtain more robust results through further in vivo or in vitro experimental analyses.

## Conclusions

In summary, our study reveals the heterogeneity of ECs and other stromal cells in MI, concurrently indicating the role of Timp1 and Fn1 in specifying EC phenotypes and facilitating further understanding of the potential value of key networks currently associated with MI progression.

### Supplementary Information

**Additional file 1.**

## Data Availability

The datasets used and/or analyzed during the current study are available from the Gene Expression Omnibus (GEO) database (https://www.ncbi.nlm.nih.gov/geo/), accession numbers: GSE136088 and GSE2329.

## Abbreviations

MI: Myocardial infarction
scRNA-seq: Single-cell RNA-sequencing
ECs: Endothelial cells
DEGs: Differentially expressed genes
CFs: Cardiac fibroblasts
GEO: Gene Expression Omnibus
PCA: Principle component analysis
tSNE: t-distributed stochastic neighbor embedding
GSEA: Gene set enrichment analysis
ECL: Enhanced chemiluminescence
HUVECs: HUMAN umbilical vein endothelial cells
