# Supplementary material for: Single-cell RNA sequencing reveals hub genes of myocardial infarction-associated endothelial cells
Source: BMC Cardiovasc Disord. 2024 Jan 24;24:70. doi: 10.1186/s12872-024-03727-z (PMC10809747; doi:10.1186/s12872-024-03727-z)

Timp1 Fig. 7a


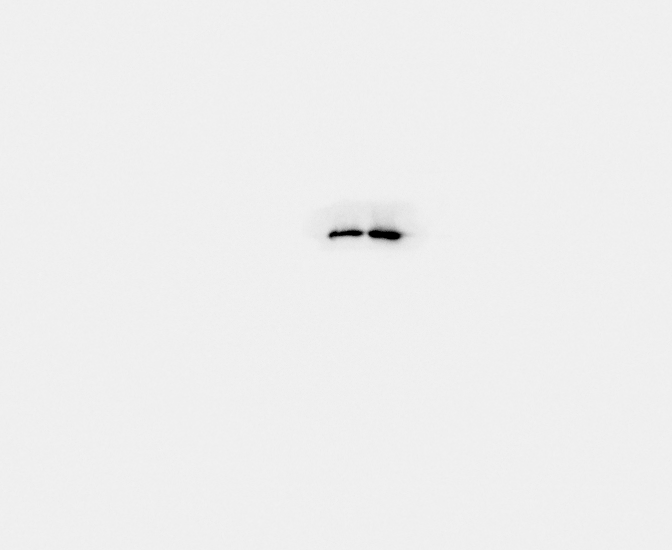

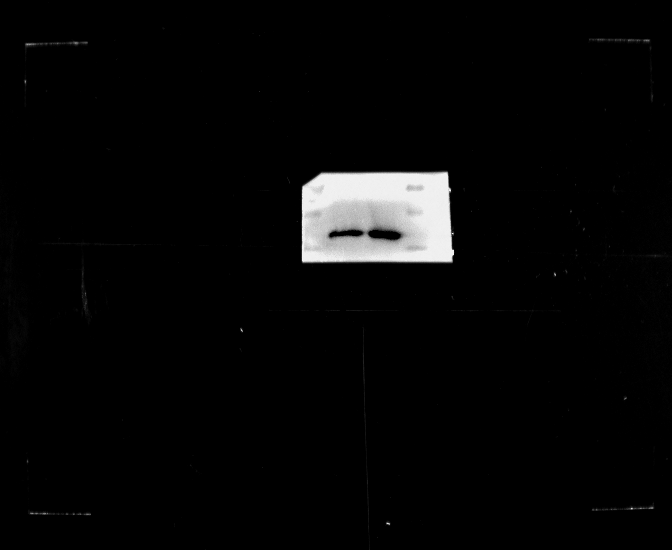


Fn1 Fig. 7a


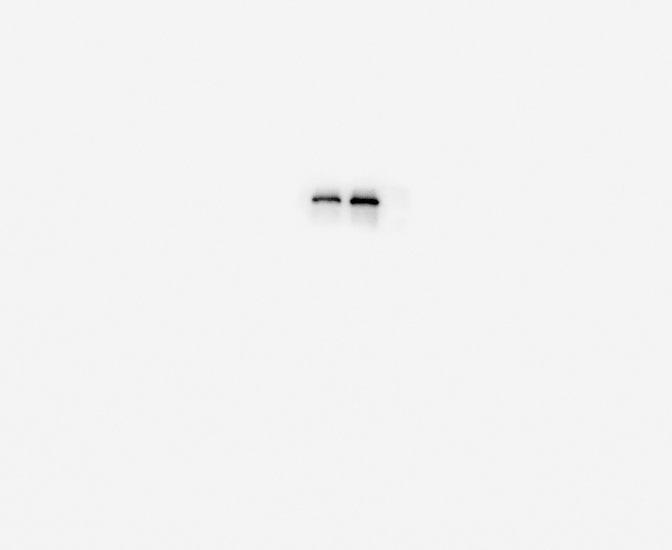


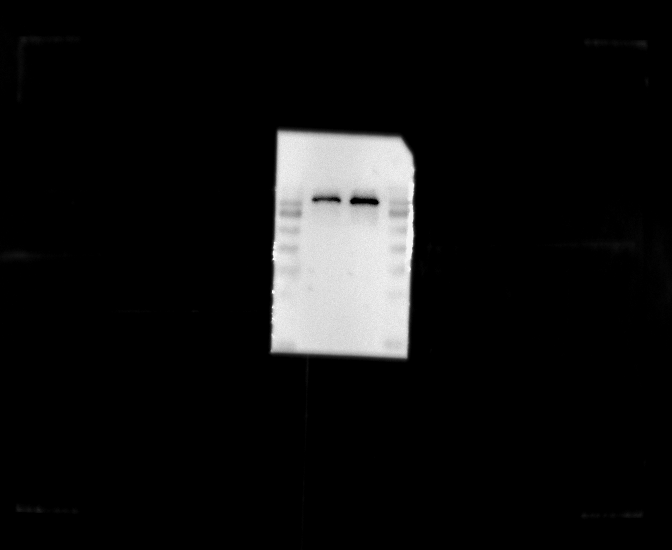


GAPDH Fig. 7a


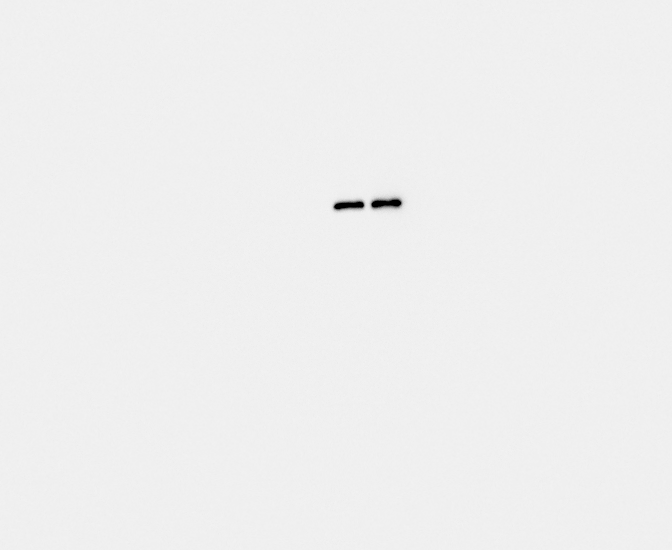

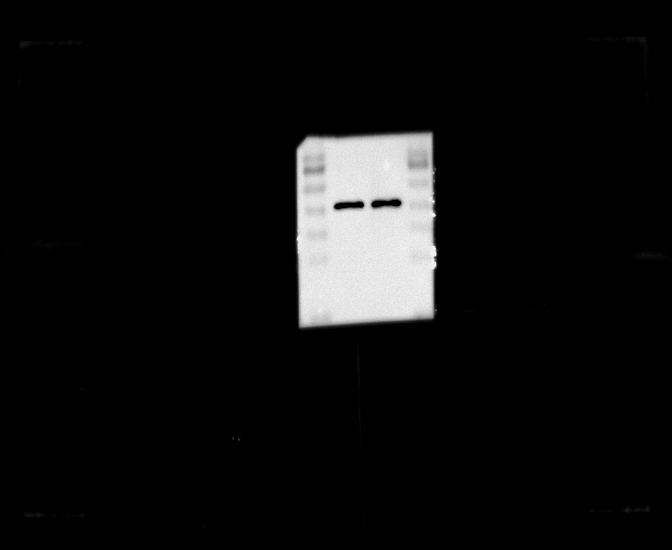


Fn1 Fig. 7b


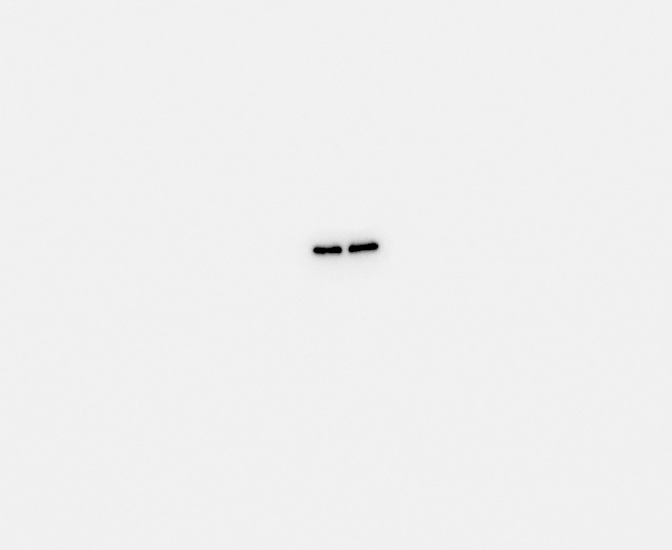

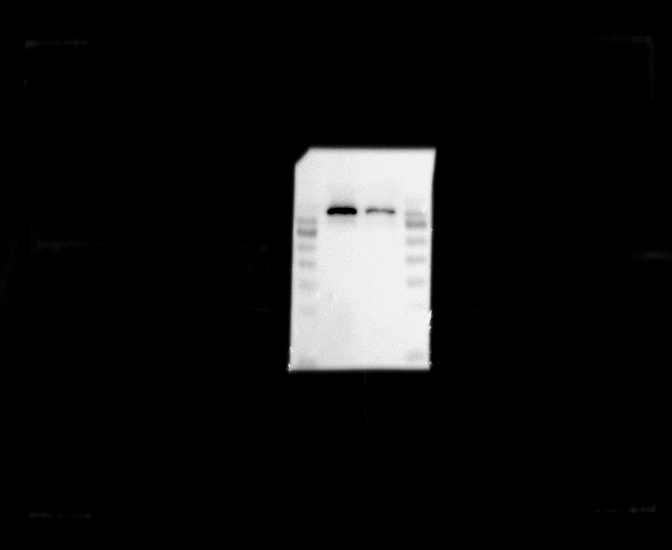


GAPDH Fig. 7b


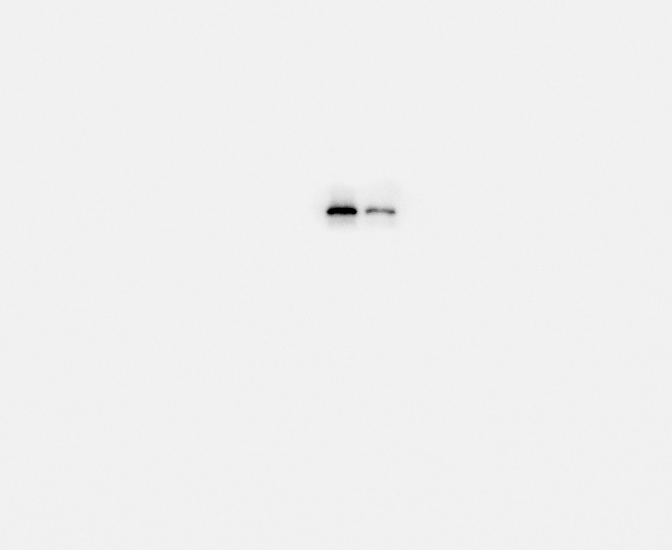

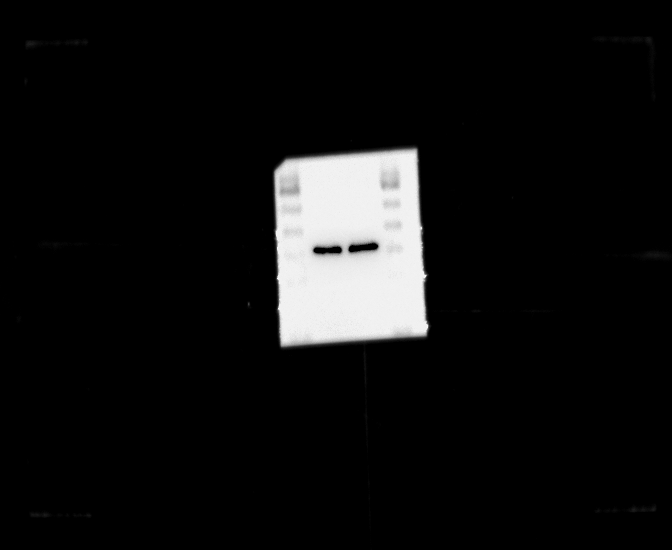


Timp1 Fig. 7b


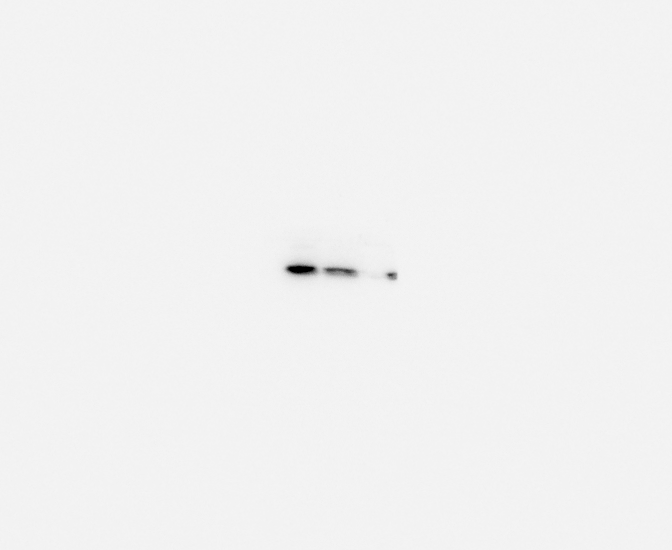

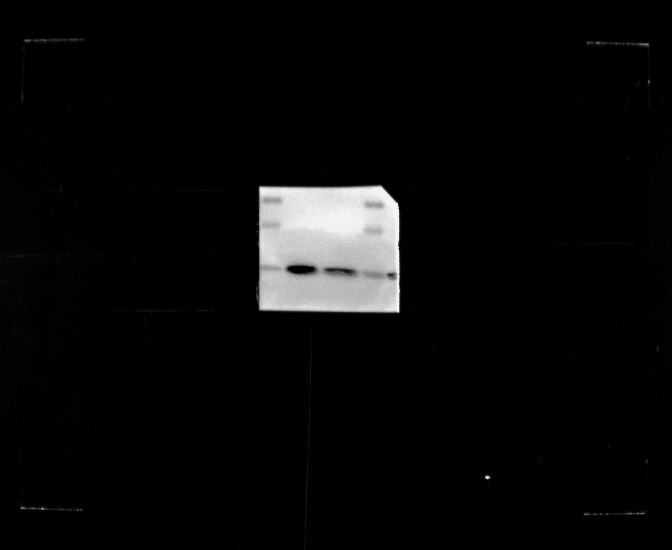


GAPDH Fig. 7b


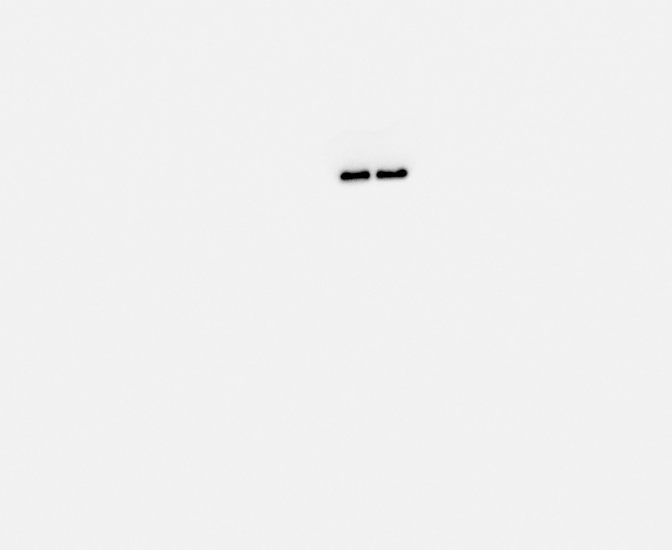

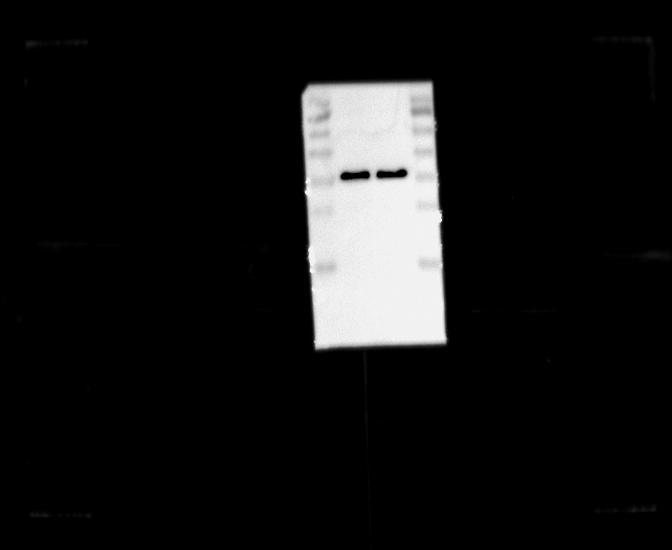


NC siRNA


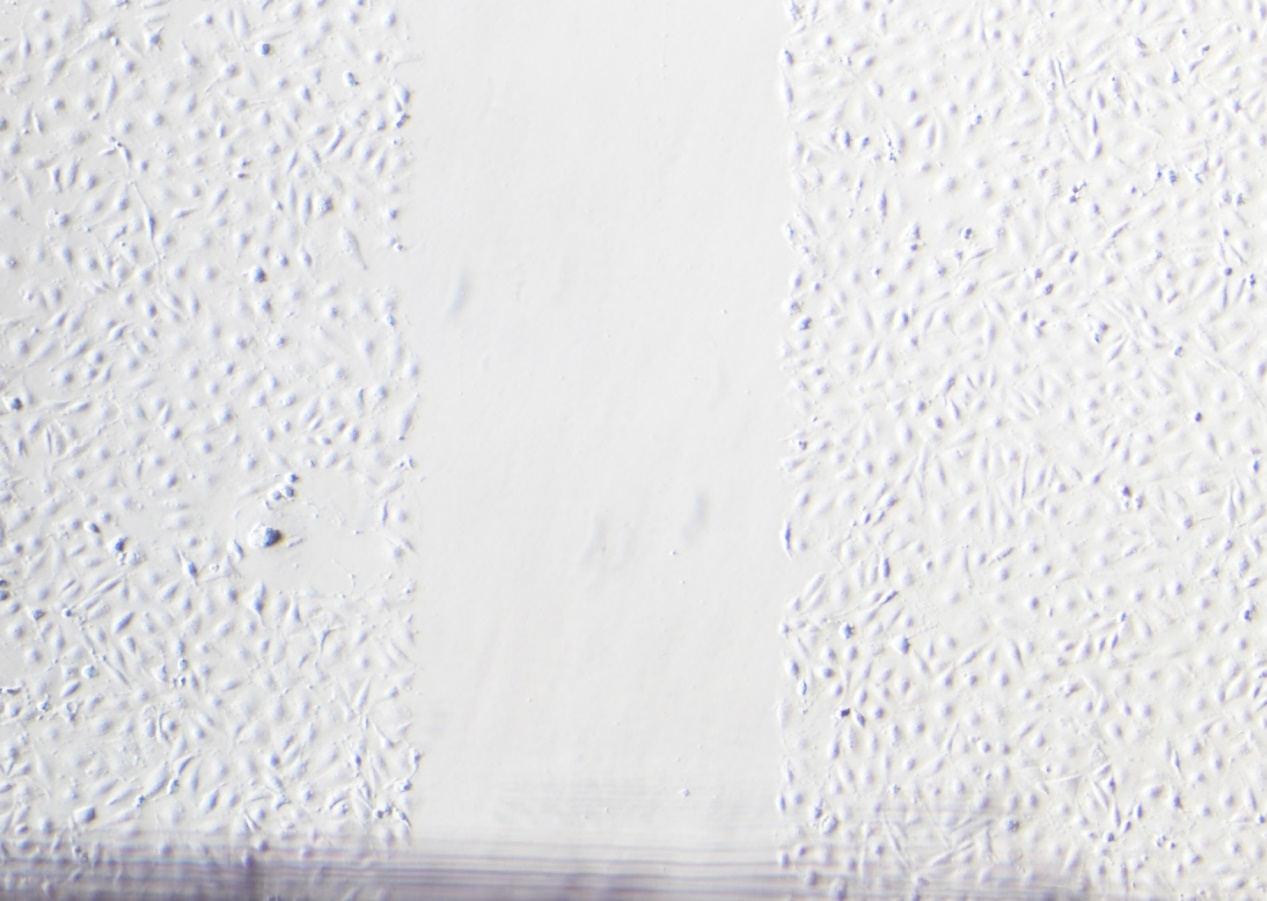

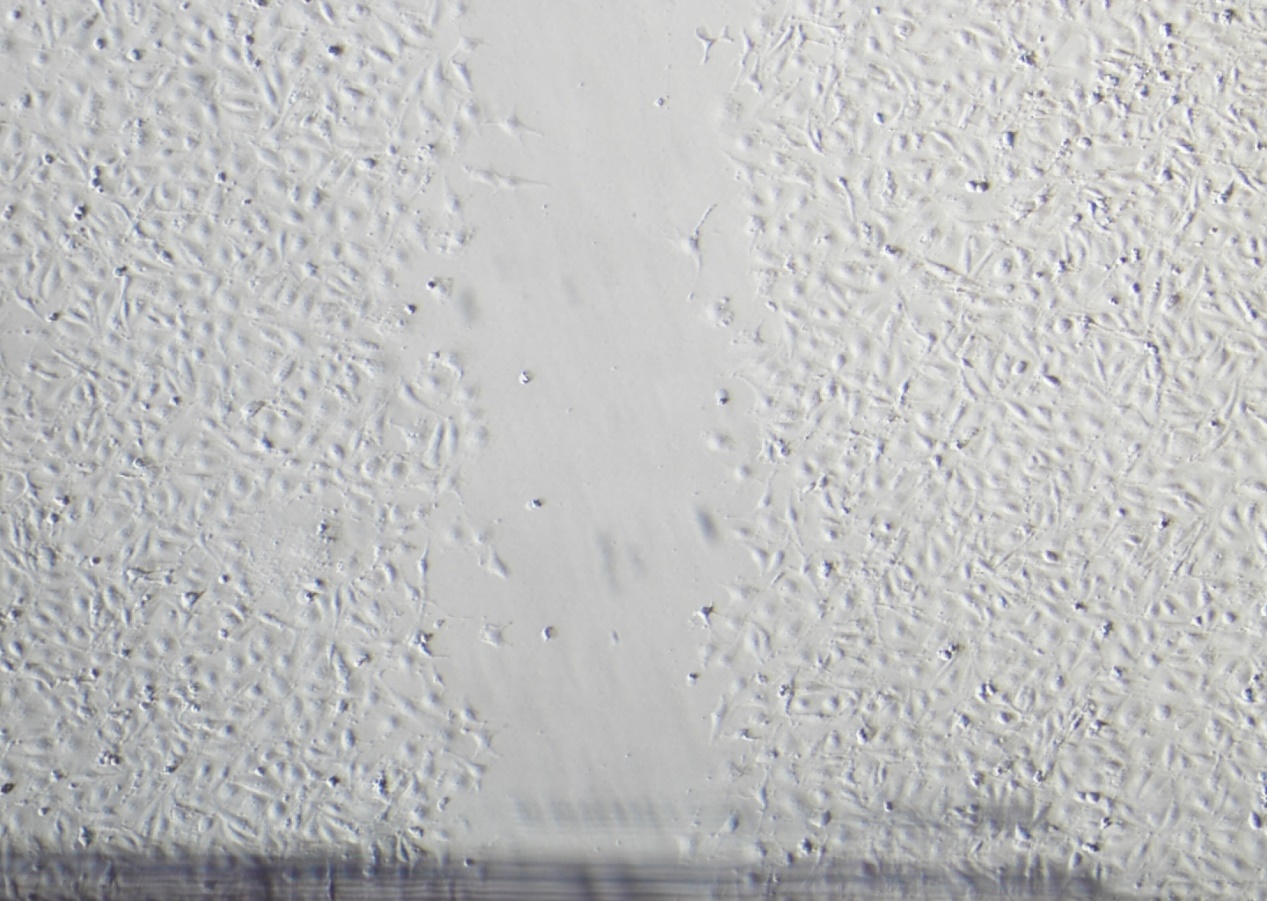


Fn1 siRNA


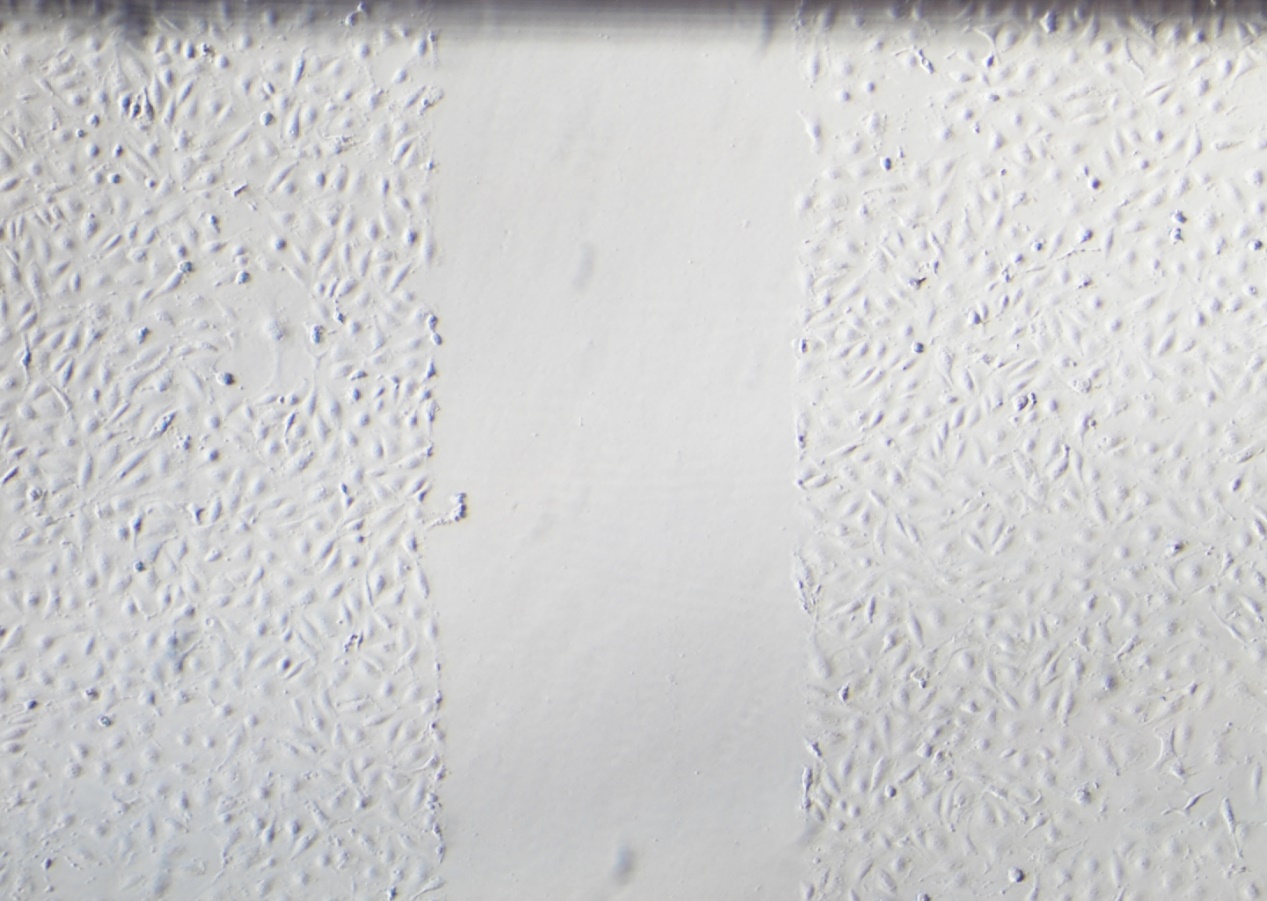

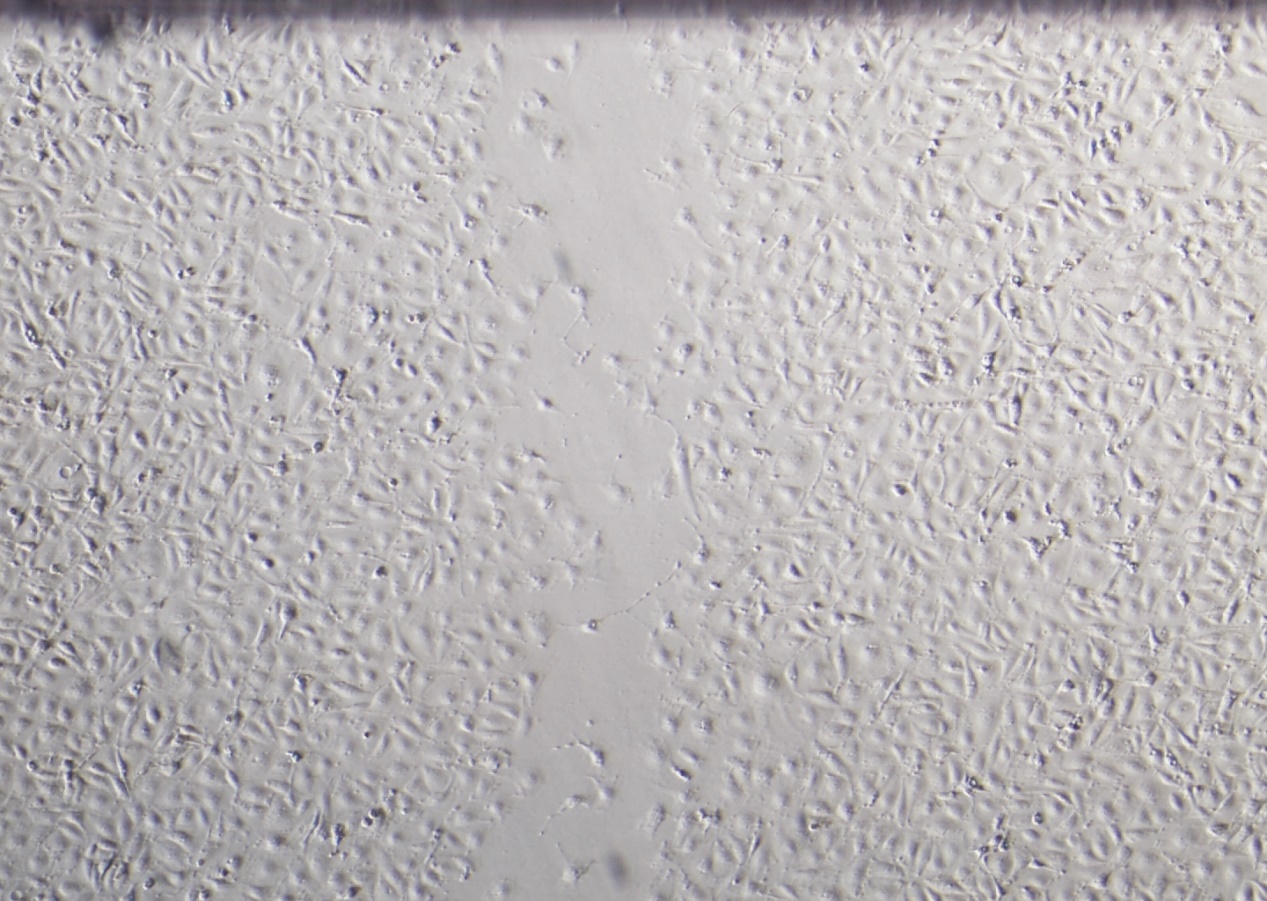


Timp1 siRNA


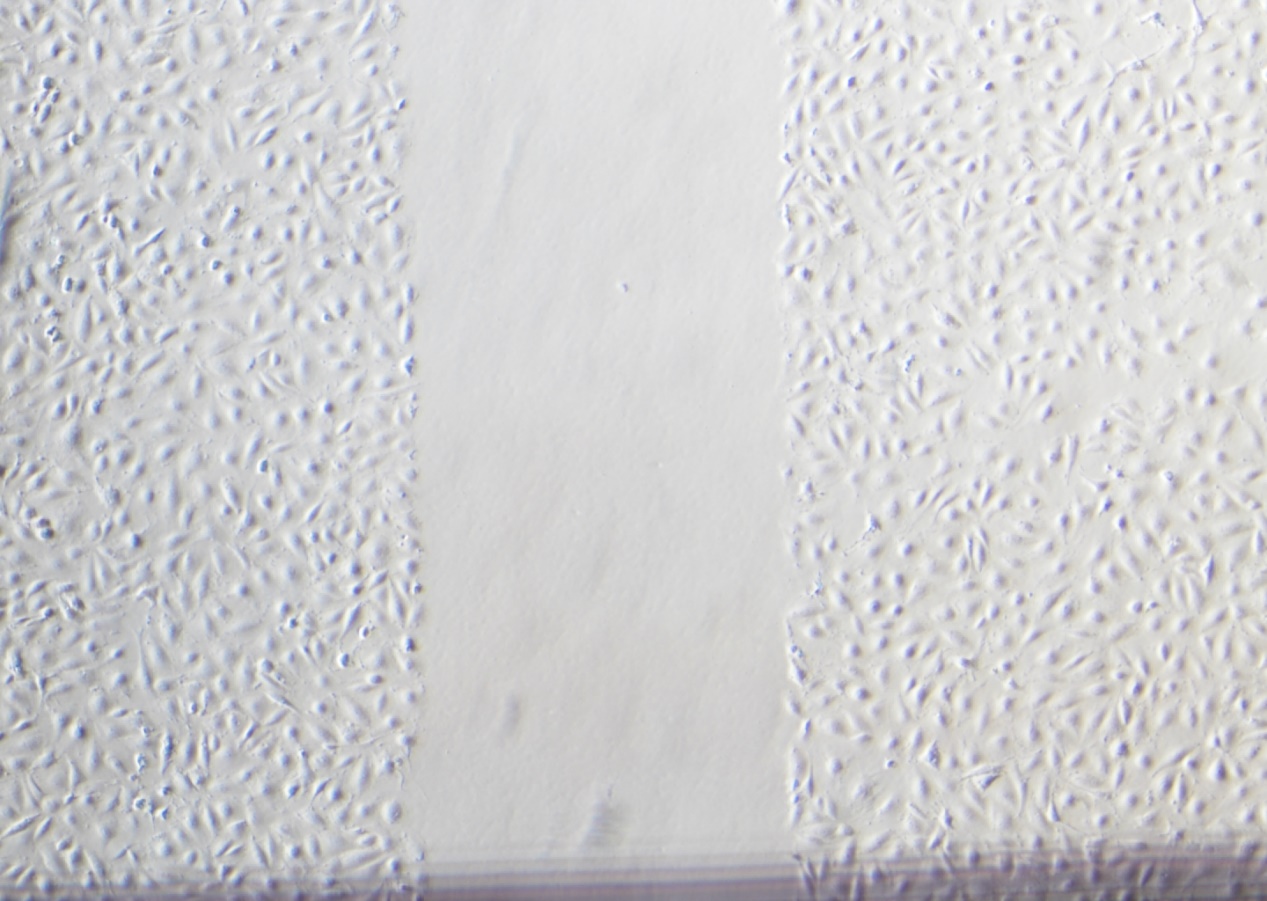

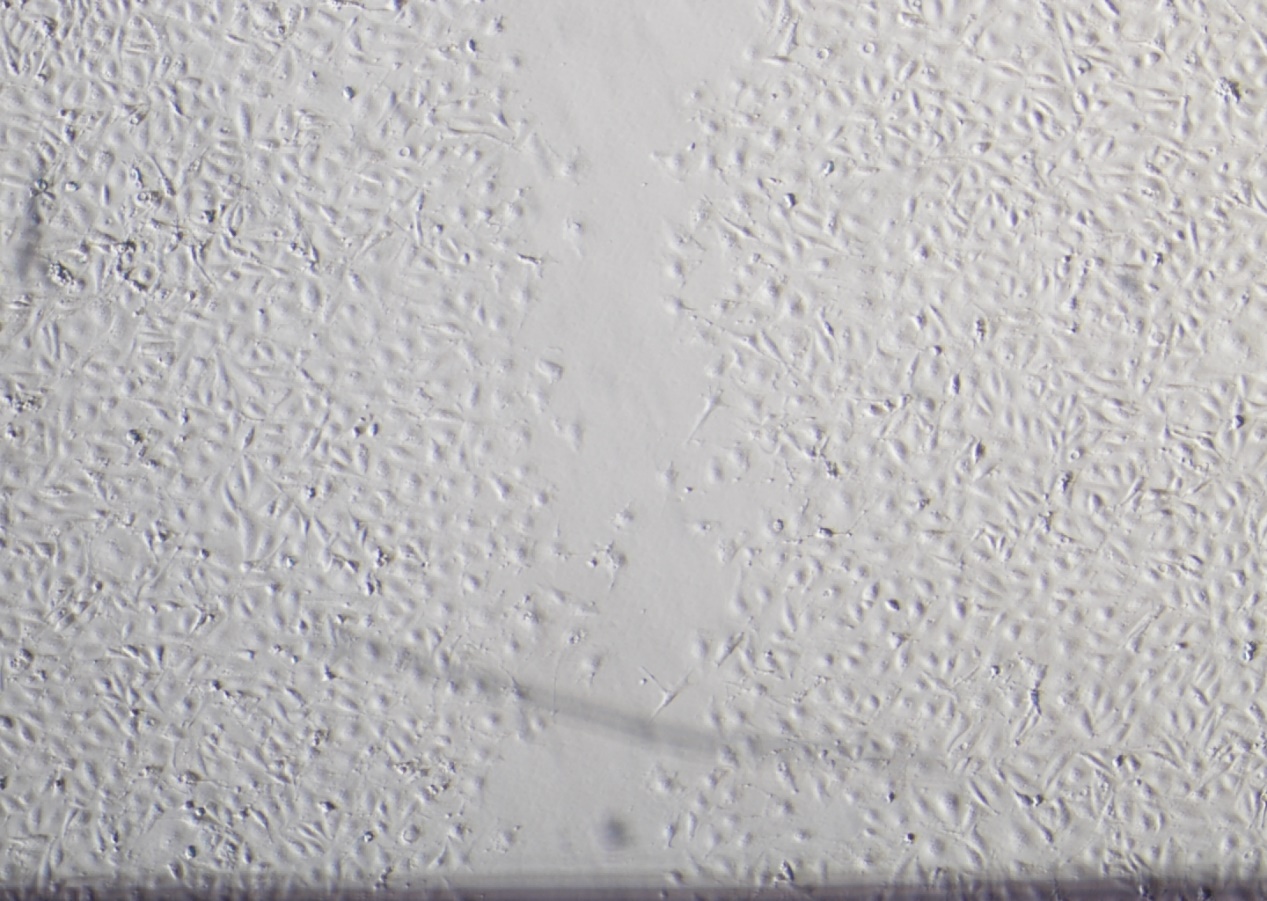


NC siRNA


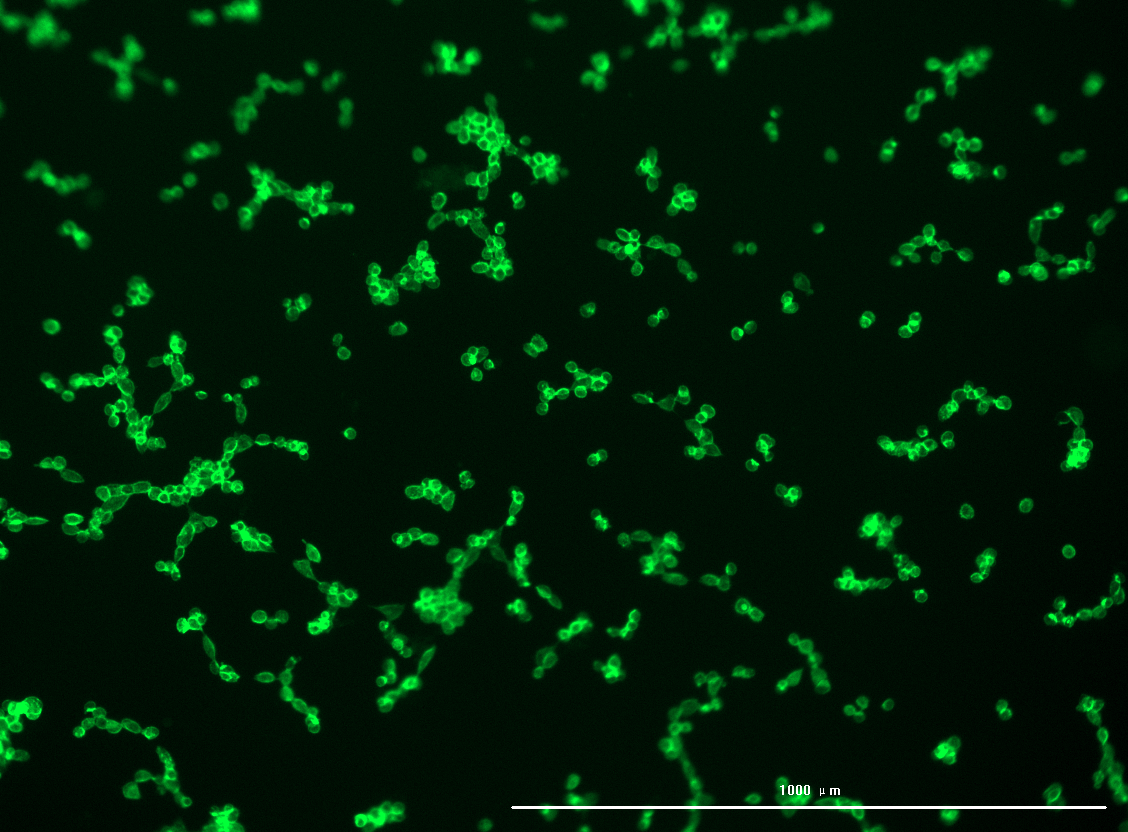


Fn1 siRNA


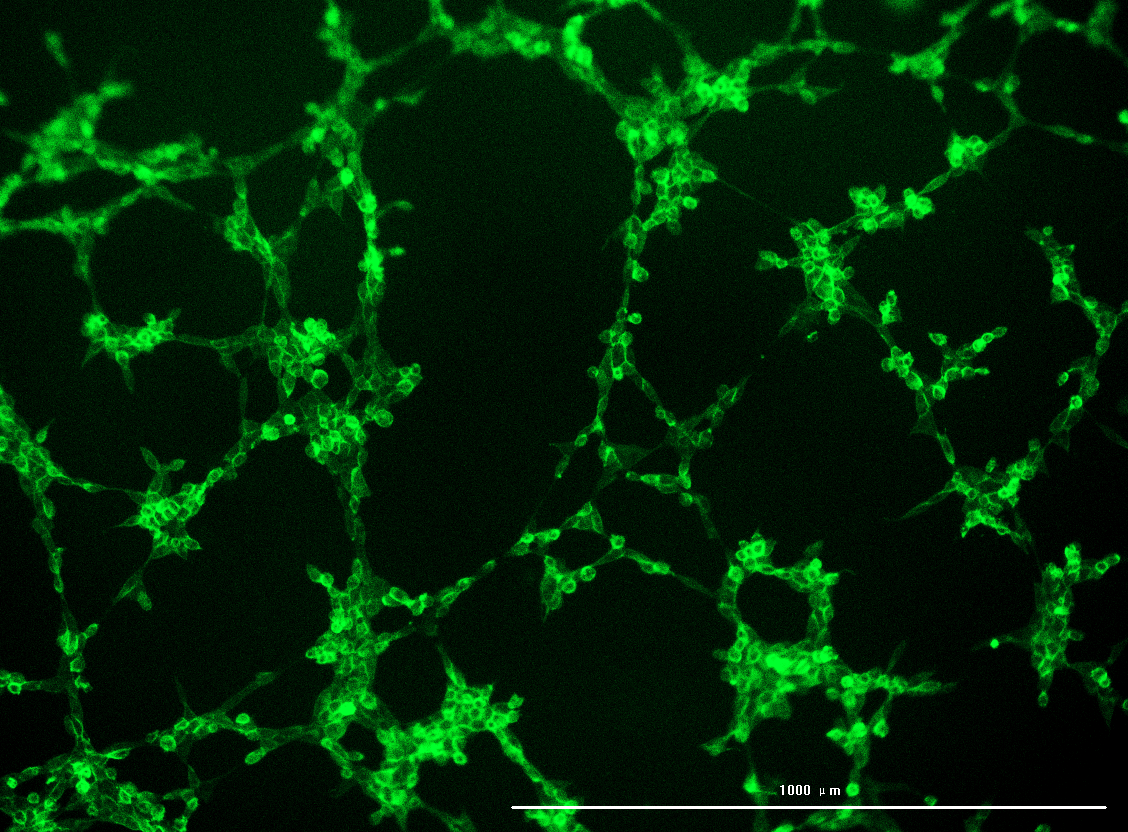


Timp1 siRNA


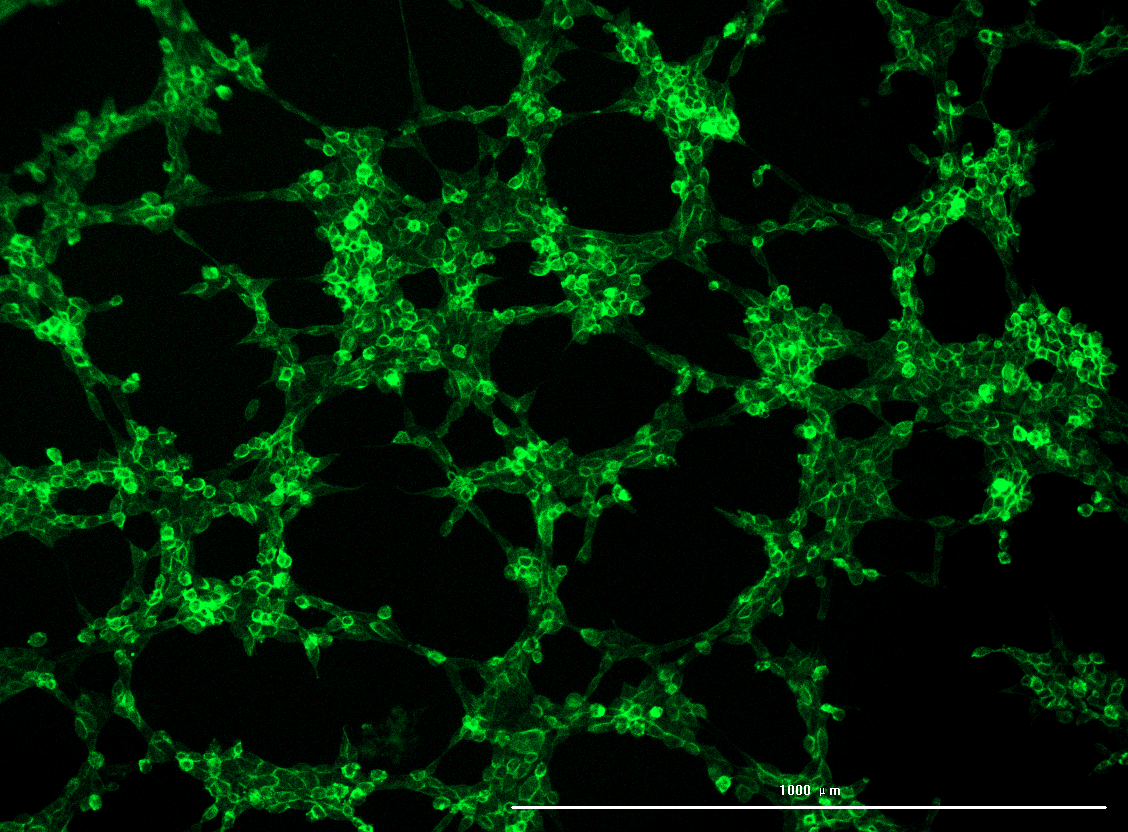

Supplement: Supplementary file 1 — Additional file 1. [file 12872_2024_3727_MOESM1_ESM.docx]
